# Probable Vancomycin Induced Neutropenia: A Case Report

**DOI:** 10.7759/cureus.5199

**Published:** 2019-07-22

**Authors:** Anahat Kaur, Ghazal Khan, Punita Grover, Jill Moormeier

**Affiliations:** 1 Internal Medicine, University of Missouri - Kansas City School of Medicine, Kansas City, USA; 2 Hematology and Oncology, University of Cincinnati, Cincinnati, USA; 3 Hematology and Oncology, University of Missouri - Kansas City School of Medicine, Kansas City, USA

**Keywords:** vancomycin, neutropenia

## Abstract

Vancomycin has been used for a long time to manage resistant gram-positive bacterial infections. Neutropenia is an uncommon and potentially serious adverse effect associated with vancomycin use. Herein we present a case of probable vancomycin induced neutropenia which resolved with discontinuation of the antibiotic. Since blood counts are monitored routinely these days in both outpatient and inpatient setting, due consideration needs to be given to vancomycin induced neutropenia in patients who are on long term antimicrobial therapy.

## Introduction

Vancomycin has been used clinically for decades to treat gram-positive infections, particularly those caused by methicillin-resistant staphylococci and for patients with severe penicillin allergies. Adverse effects from vancomycin are rare and neutropenia with vancomycin therapy is uncommon.

## Case presentation

We present a case of a 64-year-old male who was admitted with a chief complaint of fever. The patient had recently been discharged from hospital one month ago after the inpatient stay for renal failure due to sepsis and had been started on hemodialysis (HD). On admission, the patient had a temperature of 102.7 F and was tachycardic. Initial labs revealed markedly decreased white blood cell (WBC) count of 500 cells/mm^3^ (differential with 0% neutrophils, 0% bands, 0% eosinophils, 97% lymphocytes, 2% monocytes and 1% basophils). Last known baseline of WBC count was 11,900 cells/mm^3^ two weeks ago at the time of last hospital discharge. Computerized tomography scan of the chest showed bilateral ground-glass opacities and he tested positive for influenza A.

The patient was admitted to the intensive care unit for leukopenia and fever. He was started on empiric antibiotic therapy with intravenous (IV) vancomycin, piperacillin-tazobactam, and levofloxacin. He was also started on oseltamivir for influenza. Legionella sputum culture collected due to the history of uncompleted Legionella treatment during prior hospitalization came back positive. On Day 2, the patient reported diarrhea and tested positive for *Clostridium difficile* so was started on oral vancomycin for *C. difficile* coverage. On this day, IV antibiotic regimen was de-escalated to levofloxacin (for Legionella) and vancomycin only. Piperacillin-tazobactam was discontinued due to known adverse reaction of granulocytopenia, however absolute neutrophil count (ANC) did not improve after this change and remained at zero. Peripheral smear showed normocytic normochromic anemia, severe neutropenia and rare circulating blast cells. Hematology service was consulted for neutropenic fever and recommended starting Granulocyte colony-stimulating factor (G-CSF). At this point cause of neutropenia was most likely thought to be medication or sepsis-induced however bone marrow biopsy was performed to rule out other etiologies. Biopsy results showed marrow cellularity at 20-30% with preserved megakaryopoiesis. Myeloid to erythroid ratio was 0.2:1. Myelopoiesis showed maturation with the relative increase in eosinophils, basophils and mast cells. Few neutrophils were identified. There was no evidence of malignancy. Autoimmune disease panel came back negative. Blood cultures did not show any growth so, IV vancomycin was also discontinued on Day 5. 

Despite these medication changes, the patient continued to remain neutropenic and developed intermittent fevers with negative blood and urine cultures throughout admit. There was a concern for vancomycin induced neutropenia at this point since the patient had been treated with vancomycin during prior admission for sepsis (last known exposure to vancomycin was 24 days before patient’s current presentation). Although the patient remained only on oral vancomycin for *C. difficile *at this time (which has poor bioavailability), this was also eventually discontinued on Day 12 due to the possibility of contribution to neutropenia. 

**Figure 1:**
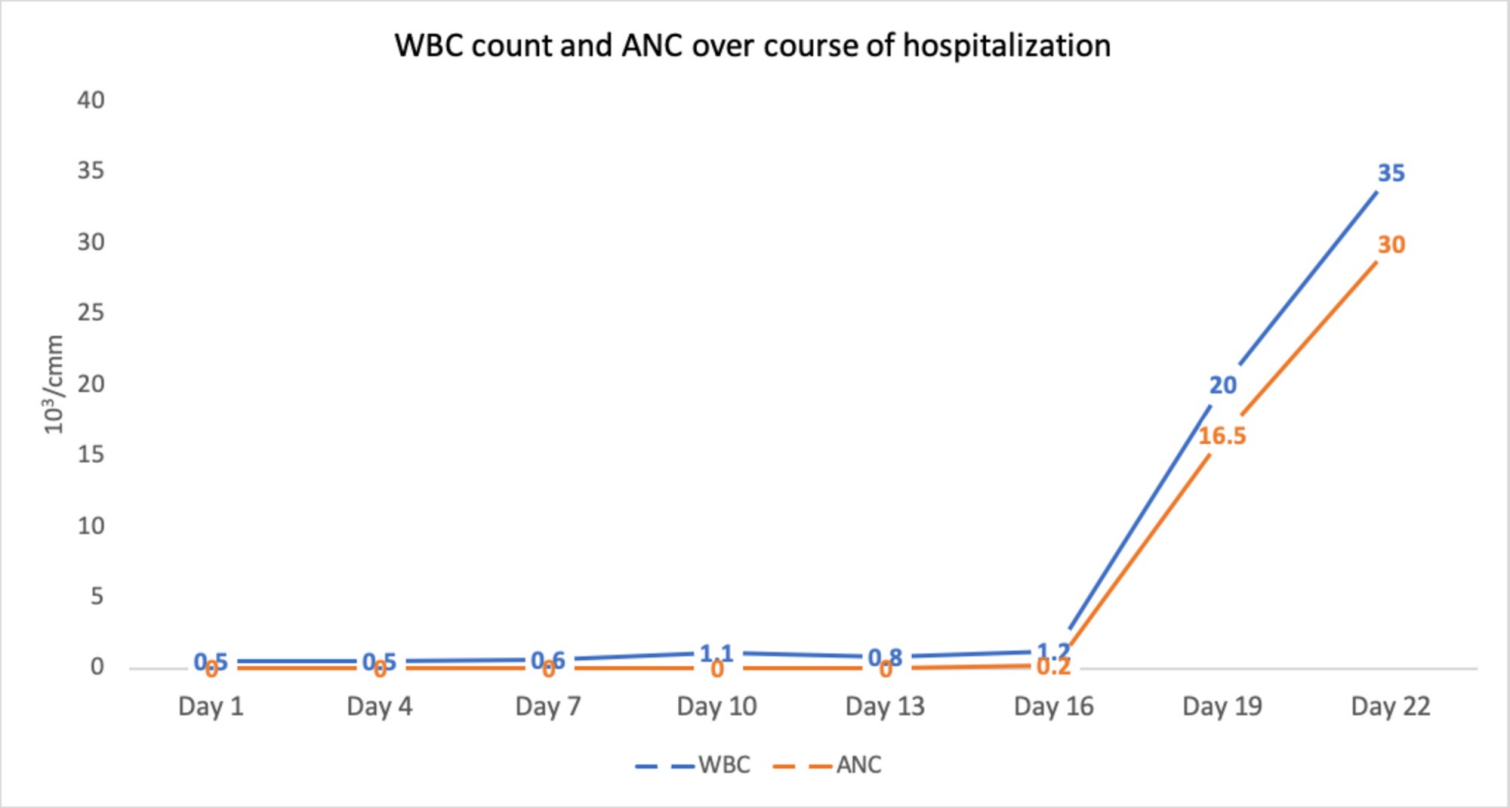
Patient's WBC and ANC count throughout the course of hospitalization. Intravenous vancomycin was discontinued on Day 5 and oral vancomycin was discontinued on Day 12. WBC - White Blood Cell, ANC - Absolute Neutrophil Count

Patient's cell counts began to improve dramatically after completely stopping vancomycin therapy (Figure 1). G-CSF was discontinued on Day 19 when WBC count went up to 20,500 cells/mm^3^. His renal function improved throughout admit and he was able to come off dialysis. The patient was eventually discharged to a rehabilitation facility and was lost to follow up.

## Discussion

Vancomycin is a glycopeptide antibiotic with reliable activity against many gram-positive pathogens and is frequently prescribed for patients with methicillin-resistant Staphylococcus aureus (MRSA) infections [1]. Its use is however associated with several adverse events, including allergic reactions, neutropenia, nephrotoxicity, ototoxicity and red man syndrome [2-6]. Neutropenia is commonly defined as ANC <1000 cells/mm^3^, severe neutropenia is defined as ANC <500 cells/mm^3^and leukopenia is defined as WBC count <3000 cells/mm^3^.

Previously reported incidence of vancomycin induced neutropenia has been between 2% to 12% [7]. Reports of vancomycin-induced neutropenia date back several decades but appear to be more common starting in the mid-1970s [8]. Incidence is similar across genders and age groups [9]. The underlying mechanism behind this remains unclear however isolation of anti-neutrophil antibodies suggests an immune-mediated event rather than direct destruction of the bone marrow [8, 10]. Examination of bone marrow biopsy specimens from patients with vancomycin-induced neutropenia has shown both hypoplasia and hyperplasia of the granulocyte series. Rapid recovery of granulocytes after stopping vancomycin therapy or administration of G-CSF suggests that myeloid precursors are likely intact [11]. The lack of a dose-dependent effect on WBC count argues against a direct toxic effect of vancomycin upon the bone marrow [9]. 

Usually, the development of neutropenia has been recognized after the first week of glycopeptide antibiotic therapy [7]. Nadir of the neutrophil count is observed 15-40 days after initiation of vancomycin and spontaneous recovery of neutrophils after cessation of antibiotics may take as long as one to 22 days with a median of six days [11, 12]. In our patient, neutropenia was present at the time of hospital admission and he was last known to have been exposed to vancomycin about 24 days ago during a prior hospitalization. Neutropenia generally does not correlate well with vancomycin dose or serum drug concentrations however compromised renal function in our patient could possibly have played a role in prolonged clearance of vancomycin [9]. Influenza and Legionella infections were believed to be unlikely causes of neutropenia in the setting of a normal platelet count. Other infectious causes of neutropenia were also felt to be less likely given the short duration of his illness and the fact that the patient clinically improved while his white count lagged behind. Hence, bone marrow biopsy was undertaken to rule out any potential malignancy which turned out to be unremarkable. Piperacillin-tazobactam was the first antibiotic to be discontinued as beta-lactams are also known to cause isolated profound granulocytopenia with rapid recovery within days once a drug is stopped, however, this did not improve ANC in our patient [13-16]. Eventual discontinuation of vancomycin had a profound effect on ANC. Based on the Naranjo adverse drug reaction probability scale, we suspect that this is a case of probable vancomycin induced neutropenia.

The first step in the management of suspected vancomycin induced neutropenia would include discontinuation of the drug. Teicoplanin, another glycopeptide antibiotic with somewhat different safety profile as compared to vancomycin, has been used as a substitute in some studies [17]. A meta-analysis of the combined results from 11 clinical trials indicated that teicoplanin was less likely to be associated with adverse effects than vancomycin [3]. Some case reports of cross-reactions to both vancomycin and teicoplanin, including neutropenia, have been published [18]. In a study conducted by Hung et al. four out of eight patients who experienced vancomycin induced neutropenia subsequently experienced teicoplanin induced neutropenia as well [7]. The use of teicoplanin in patients with vancomycin-related adverse effects is controversial [7, 18]. In situations where continuation of uninterrupted vancomycin therapy is imperative despite neutropenia (patients with penicillin allergy), another option would be to consider G-CSF administration [11]. The dose of G-CSF must be individualized specifically in patients requiring prolonged administration of vancomycin therapy, with a goal to maintain ANC between 1000-10,000/mm^3^. This would need intermittent dosing titrated to maintain an acceptable degree of response [11]. Re-challenge with vancomycin is generally not recommended given complications associated with neutropenia and the possibility of a more intense neutropenic reaction likely due to immune sensitization to the drug. Monitoring for vancomycin-induced neutropenia is important in patients receiving the drug for longer than two weeks duration [9].

## Conclusions

Due consideration needs to be given to vancomycin as a possible causative agent in setting of neutropenia of unclear etiology. Since complete blood count is now routinely monitored in both outpatient and inpatient setting, vancomycin-induced neutropenia is seen more often among patients who are receiving long-term antimicrobial therapy. Further management in this scenario would include discontinuation of vancomycin if considered appropriate or administration of G-CSF to maintain counts while patient completes antimicrobial therapy.
